# A proposal for changing nomenclature from pseudomyxoma peritonei (PMP) to abdomino-peritoneal mucinous carcinoma (APM) based on its long journey and experience from tertiary oncology center in India

**DOI:** 10.1186/s12957-022-02639-6

**Published:** 2022-06-01

**Authors:** M. D. Ray, Manish Kumar Gaur, Chandan Kumar, S. V. S. Deo

**Affiliations:** Department of Surgical Oncology, DR BRA-IRCH, All India Institute of Medical Sciences, New Delhi, India

**Keywords:** Pseudomyxoma peritonei (PMP), Abdomino-peritoneal mucinous carcinoma (APM), Cytoreductive surgery (CRS), Hyperthermic intraperitoneal chemotherapy (HIPEC), Appendicular neoplasm

## Abstract

**Introduction:**

Pseudomyxoma peritonei (PMP) is a generalized term, usually known as “jelly belly” since 1884. Incidence is very low, 1–3 per million people per year. Because of its indolent nature, it is usually diagnosed at an advanced stage, thereby impacting the quality of life. The 5-year survival rate varies from 23 to 86% in world literature. Even 10 years and 20 years of survival have been described. With our experience, we like to propose rename of PMP as abdomino-peritoneal mucinous carcinoma (APM) as we strongly feel the time has come to specify the term and standardize the management strategy.

**Methodology:**

In the premier institute of India and as a tertiary referral center, we experienced the maximum number of advanced cases of APM. From 2012 to 2021, we analyzed all the APM patients based on a prospectively maintained computerized database in the department of surgical oncology and found the reasons for renaming from this traditional one.

**Results:**

We included a total of 87 patients who underwent surgical intervention. Thirty-five patients underwent cytoreductive surgery (CRS) with hyperthermic intraperitoneal chemotherapy (HIPEC), and 52 patients underwent debulking. In CRS-HIPEC patients, CC-0 was achieved in 28 patients (80%), CC-1 in 4 patients (11.4%), and CC-2 in 3 patients (8.6%). Palliative intent HIPEC was done in 3 patients (8.6%). Clavien-Dindo grade III and IV morbidity was observed in 18.8% of patients with 90 days mortality of 5.7%.

**Conclusion:**

With our long-term experience and advancement of scientific evidence, we like to propose a new name for PMP as APM. We strongly believe this paper will give a clear picture of this rare disease and standard management outlines.

## Introduction

Pseudomyxoma peritonei (PMP) is a rare clinical condition with an incidence of 1–3 million per year and is characterized by mucinous deposits in the peritoneal cavity [1]. It is a generalized term and loosely known as “jelly belly” since first described by Carl F. Rokitansky in 1842 [2]. The term pseudomyxoma peritonei was coined by Werth in 1884 while describing a case of carcinoma ovary with mucinous ascites. Because of its indolent nature, it is usually diagnosed at an advanced stage, thereby impacting the quality of life. Five-year survival varies from 53 to 75% in literature [2]. Even 10 years and 20 years of survival have been described. The majority of PMP originates in the appendix. In 1901, Frankel described the first case associated with a cyst of the appendix, the primary source of pseudomyxoma peritonei [3]. The term pseudomyxoma peritonei syndrome has gradually come out, which is defined as the distribution of free or organized mucin in the peritoneal cavity, either cellular or acellular, with typical redistribution phenomena [4]. Why this kind of wide variation in survival in different literature? We tried to analyze the reason based on our experience and literature review in PubMed, Google Scholar, Medline, EMBASE, and others. With our long experience, we like to propose rename of pseudomyxoma peritonei (PMP) as abdomino-peritoneal mucinous carcinoma (APM) as we strongly and logically propose that time has come to specify the term and thereby standardize management.

### Etymology and controversies

PMP was initially described as a primary peritoneal disease with free or organized mucin in the peritoneal cavity. Myxomas are benign tumors of connective soft tissue most commonly seen in the heart. The term “pseudomyxoma” is not based on the histological feature of myxoma, but its etymology comes from pseudomucin, with the Latin word “myxo” derived from the Greek word “muxa” meaning “mucus” [5]. We now know that PMP is neither a real myxoma nor a primary peritoneal disease but occurs due to peritoneal dissemination commonly from primary tumors of appendicular origin. Apart from this, there are different mucin-producing organs like the intestine, gall bladder, pancreas, mature teratoma ovary, endocervix, urachus, breast, and lung. Traditionally, PMP is commonly used for many diseases, and a sound definition is still not available [1]. Sometimes, the term is loosely reported for any condition with mucinous ascites [6].

### Etiopathogenesis

Although multiple abdominal organs containing goblet cells can produce mucin, the appendix is the primary organ in the majority of cases. A primary mucinous tumor develops following the neoplastic transformation of goblet cells forming mucocele [7]. Peritoneal dissemination of the mucin occurs after rupture of these mucoceles with the deposited mucinous cells keep secreting mucin in the peritoneal cavity [8]. With time, these mucinous deposits may increase in volume, increase intra-abdominal pressure, even compress the different viscera, and lead to bowel obstruction. The classical deposit site is best described by the “redistribution phenomenon” with the movement of free-floating epithelial cells by gravity and concentration at the sites of peritoneal fluid absorption. Tumor deposits are commonly found both on greater and lesser omentum and under the right side of the diaphragm, but typically, the tumors are absent on the intestine's surface, which may be because of peristaltic activity [9]. The concept of the ovary as a primary organ of PMP is refuted today [10]. However, PMP can infrequently arise from mature ovarian teratoma, which is the only reported etiology of PMP from the ovarian primary [11, 12].

### Classification of PMP

Since 1884, the term PMP has been prevailing worldwide. In 1995, however, Ronnett et al., for the first time, tried to correlate the histology of PMP with prognosis. They classified PMP as (i) Diffuse Peritoneal Adeno Mucinosis (DPAM), which is indolent in the course and without any invasion, (ii) Peritoneal Mucinous Adeno Carcinoma (PMCA), which is more aggressive as it contains a higher percentage of malignant cells and (iii) Peritoneal Mucinous Adeno Carcinoma Intermediate (PMCA-I) which is hybrid, as it has the features of both DPAM and PMCA [13]. After that, Misdraji et al. found that in 52 months follow-up, the DPAM has 96% disease-free survival (DFS) where only 33% DFS was observed in PMCA [14].

WHO classified PMP as low-grade appendicular mucinous neoplasia (LAMN) and high-grade appendicular mucinous neoplasia (HAMN). As low grade, they defined mucinous pools with low-grade dysplasia with 5 years survival of 83%. In comparison, the high-grade mucinous neoplasm was defined as mucin pools with high-grade dysplasia associated with rupture and spillage throughout the abdominal cavity [15]. HAMN is associated with 5 years survival of 68%.

According to the Peritoneal Surface Oncology Group International (PSOGI), PMP was classified histopathologically as follows: (i) mucinous tumors—when >50% of the field through microscopic view contains mucus and no cancer cells; (ii) cellular mucin >50% of the high power field contains mucin with cancer cells, recurrence rate 33–77%; (iii) acellular mucin <10% mucin of the field with cancer cells, recurrence rate 3–7% only; (iv) PMP with low-grade histologic features—low-grade mucinous carcinoma peritonei or DPAM; (v) PMP with high-grade histologic features—high-grade mucinous carcinoma peritonei or PMCA; and (vi) PMP with signet ring cells—high-grade mucinous carcinoma peritonei with signet ring cells or peritoneal mucinous carcinomatosis with signet ring cells (PMCA-S). So, as per PSOGI, no PMP should be considered benign [16, 17].

## Methods

In the premier institute of India and as a tertiary referral center, we managed 87 advanced cases of APM. Since 2012–2021, we analyzed all the patients of APM based on a prospectively maintained computerized database in the department of surgical oncology and found the reasons for renaming from this traditional one.

### Approach to management of APM

#### Diagnostic approach

**Fig. 1:**
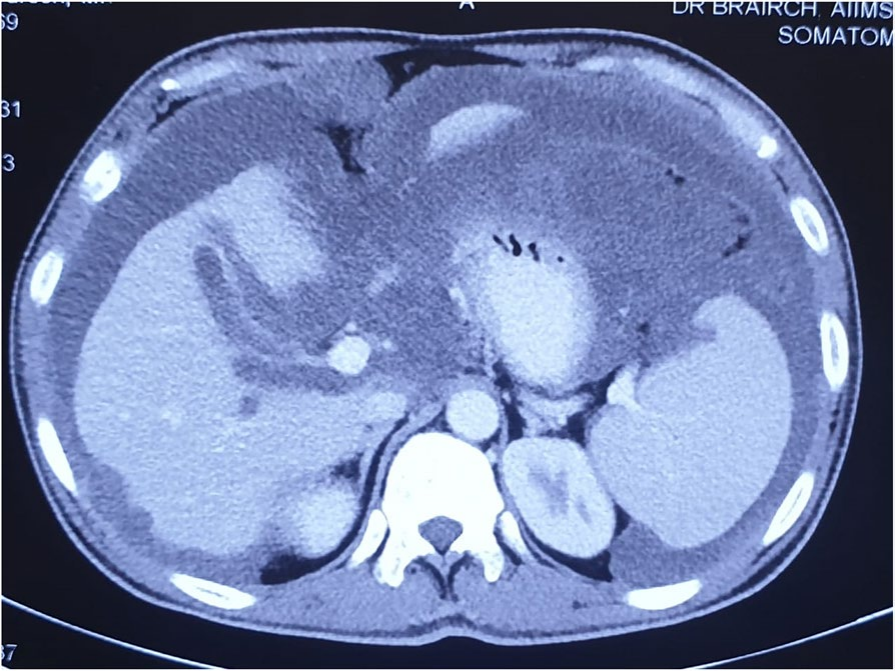
CT scan showing ascites with liver and spleen scalloping

We like to perform CECT whole abdomen along with the clinical features. The following characteristics indicate the APM: (i) curvilinear calcified mass (eggshell appearance) lesion at the ileocecal junction without any faecolith, (ii) appendicular mucocele is a low attenuation, well-capsulated round or tubular cystic mass, (iii) mass lesion more than 2 cm and absence of peri appendicular stranding more likely to be LAMN, (iv) isolated focal distal appendicular dilatation with a segment of morphologically normal appendix proximally—strongly associated with underlying neoplastic appendicular mucocele, (v) peri-appendicular fluid collection, and (vi) pattern of high-density mucinous patches without bowel and mesentery involvement scalloping over liver and spleen [18] (Fig. 1).

CT scan is a standard imaging modality to identify the extent of the disease. However, there are a few inherent disadvantages with CECT, like it cannot differentiate (i) between mucinous and ascitic fluid, (ii) bowel involvement either compartmental or diffuse, and (iii) subdiaphragmatic and pelvic areas are not well delineated in CECT.

#### MRI in APM

MRI scan can better differentiate between fluid and mucin. It can better delineate bowel and mesenteric involvement, subdiaphragmatic and dependent areas [19]. Few centers like the French PSM group (RENAPE) routinely use MRI for pseudomyxoma.

#### Role of PET CT

We usually do not do PET CT routinely. Nevertheless, few studies suggest that PET CT can differentiate between DPAM and PMCA. SUV of more than 2 is suggestive of poor prognosis [20].

#### Colonoscopy

Appendicular mucinous lesions may be incidentally found on colonoscopy as the smooth indentation of the caecal lumen, mass arising from the appendiceal orifice (Volcano sign), or as exudate coming out of the orifice. On probing with biopsy forceps, a tumor can be firm in consistency or soft, collapsing with a central indentation (Cushion sign) [21].

Apart from this, we routinely evaluate tumor markers like CEA, CA 125, and CA 19.9. A study suggests that the elevation of these three markers together lower both DFS and overall survival (OS) [22].

Inflammatory markers like Platelet Lymphocyte Ratio (PLR) and Neutrophil Lymphocyte Ratio (NLR) are prognostic indicators. Many Peritoneal Surface Malignancies (PSM) centers are routinely doing as the study suggests that elevated PLR was associated with the shorter OS because the platelets facilitated tumor progression by promoting proliferation, angiogenesis, and metastasis [18].

As far as Peritoneal Cancer Index (PCI) is concerned, unlike in other PSM, APM does not follow the rule. Even in high PCI, optimal cytoreductive surgery (CRS) can be achieved. Anatomical location is a more important determinant for CRS, not the PCI score. Organized large mucinous deposit (>3 cm) at porta hepatis, superior mesenteric artery origin, over inferior vena cava, retro-hepatic area, or multiple small bowel invasion may prevent performing optimal CRS [23, 24].

### Treatment strategy

In a recent paper of 2021, Shigeki Kusamura et al. described the prognostic role of Hyperthermic intraperitoneal chemotherapy (HIPEC) in APM. They studied 1924 patients (1993–2017) in two arms, with 376 patients in the CRS-only group and 1548 in the CRS HIPEC group. They concluded that HIPEC was associated with better survival in after CRS and generally without adverse effects on surgical outcomes [25]. We follow the same strategy, and our main aim is to achieve CRS and provide HIPEC in all patients. Despite no level 1 evidence, because of the rarity of the disease, CRS HIPEC is the standard treatment modality across the globe. With response to systemic therapy in APM ranging from 4 to 24%, any form of intraperitoneal chemotherapy has an immense role in preventing recurrence and increasing overall survival. Even few studies suggest debulking surgery has got a role in survival and quality of life [26–28]. Surprisingly, Sugarbaker et al. observed 20 years survival of 70% following CRS combined with intraperitoneal chemotherapy using Mitomycin at 42 °C followed by fluorouracil for 4–5 days. As per Paul Sugarbaker, complete cytoreduction is defined as leaving behind no macroscopic disease (CC-0) or macroscopic disease up to 2.5 mm (CC-1), where one should go for HIPEC.

As per the Chicago consensus, indications of CRS and HIPEC in APM include (i) LAMN, (ii) appendicular adenocarcinoma, and (iii) goblet cell carcinoma [29]. In a retrospective study of 2298 patients, Chua et al. reported 10 years survival of 63%, with a post-operative complication rate of 24% and a reoperation rate of 10% [30]. In a multi-institutional study of 1290 patients, Glehan et al. observed the 5-year survival of 73% and reported better outcomes if HIPEC was performed in high-volume centers [31].

## Results

A total of 87 patients underwent surgery for APM from 2012 to 2021 at our center. At presentation, the median age was 55 years (range 44–66 years). Of 87 patients, 56 were females (64.4%), and 31 were males (35.6%). We included 35 patients who underwent CRS and HIPEC and 52 patients who underwent debulking surgery.

Among CRS-HIPEC patients, 26 out of 35 patients (74%) had received some form of surgical intervention in past or neoadjuvant chemotherapy. Seven patients (20%) had adenocarcinoma, 3 patients (8.5%) had moderately differentiated carcinoma, another 7 (20%) had mucinous adenocarcinoma, five patients (14.3) had a signet ring cell carcinoma, 11 patients (31.4%) had LAMN, and 2 patients had DPAM. In both the patients of DPAM, the patients had undergone prior appendicectomy. CC-0 was achieved in 28 patients (80%), CC-1 in 4 patients (11.4%) and CC-2 in 3 patients (8.6%). Palliative intent HIPEC was done in 3 patients (8.6%) given recurrent gross mucinous ascites to improve the symptoms and provide a better quality of life.

In the rest 52 patients, only debulking surgery was performed. Fourteen patients were post-neoadjuvant chemotherapy. In the final pathological report, 13 patients had adenocarcinoma, 11 patients had moderately differentiated carcinoma, 15 patients had mucinous adenocarcinoma, 9 patients had signet-ring cell carcinoma, 3 patients had LAMN, and 1 patient had DPAM. The patients were labelled as DPAM when no definite focus of origin was found in the final histopathological specimen, and no malignant cells were found in pools of mucin.

In our experience, Clavien-Dindo grade III and IV morbidity was observed in 18.8% of patients with 90 days mortality of 5.7%. The most common complications in our series included post-operative ileus, surgical site infection, pleural effusion, respiratory insufficiency, bowel perforation, wound dehiscence, and anastomotic leak. Initially, pulmonary embolism and deep vein thrombosis rate was as high as 8%. With the use of low molecular weight heparin Dalteparin, 100 IU/kg body weight subcutaneously 12 h before surgery and 6 h after surgery or latest by postoperative day 1 morning, the rate of thromboembolic events had been reduced dramatically. Ninety days mortality was seen in 2 patients (5.7%)

### Our experience

#### Our technique

We know the selection is the Queen and biology is the King, but being at the premier institute, our selection is beyond our choice as most of the patients are referred to us with the last hope and for the best treatment. We start prehabilitation at the first visit when we plan forCRS. A minimal 3 weeks prehabilitation is recommended. We ask to stop smoking, alcohol, or any form of tobacco instantly maintain hydration, high protein diet, and hygiene. We also ask for incentive spirometry 20 times per hour or 200 times a day, along with exercise, yoga, and meditation. We perform a 6-min walk test on admission.

**Fig. 2:**
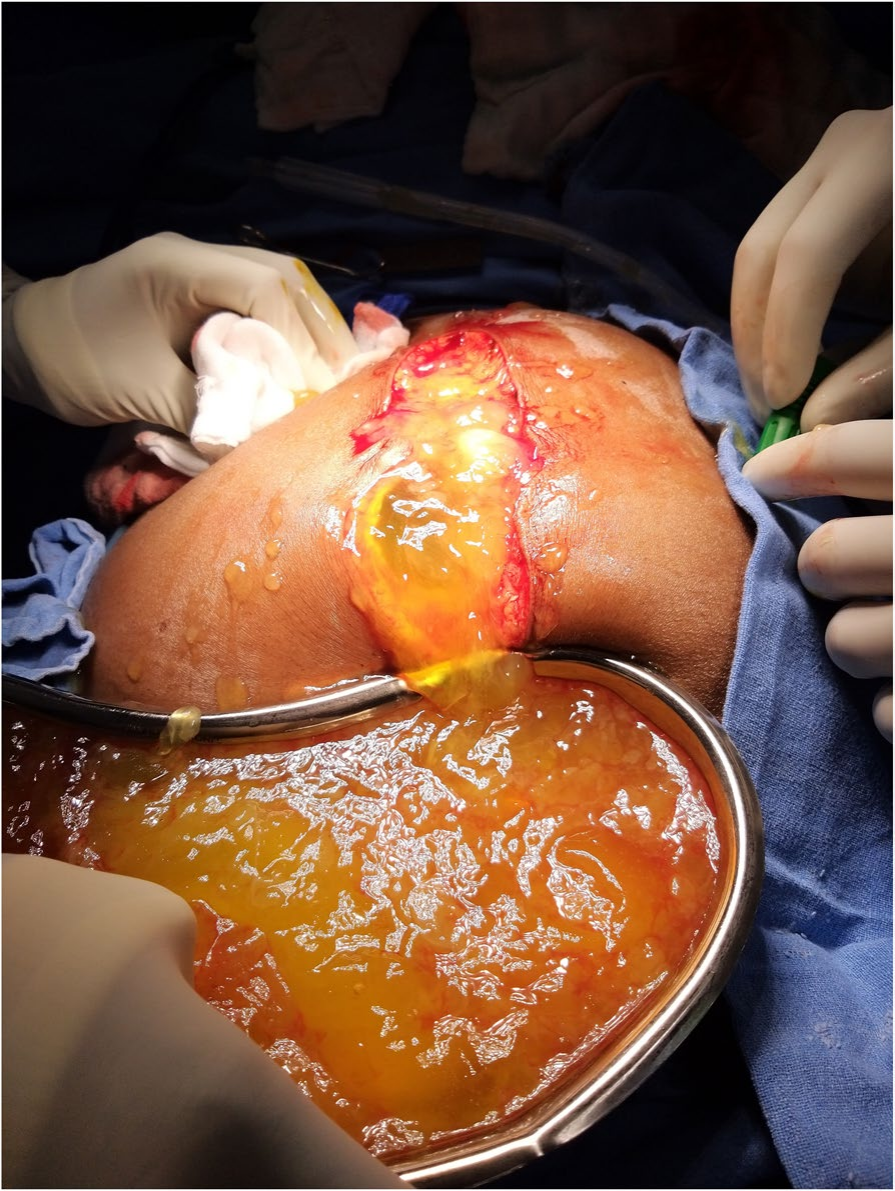
Mucinous ascites being drained on laparotomy

**Fig. 3:**
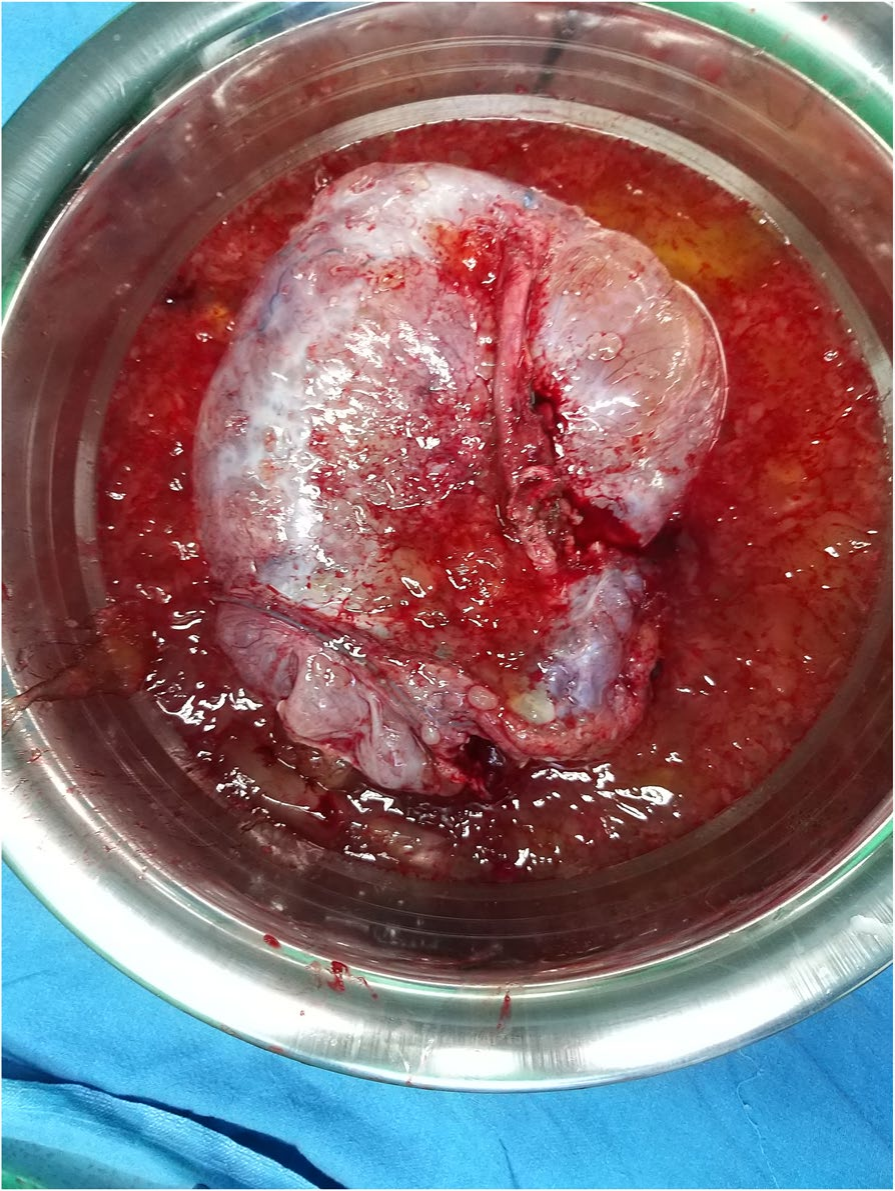
Resected specimen with mucinous content

**Fig. 4:**
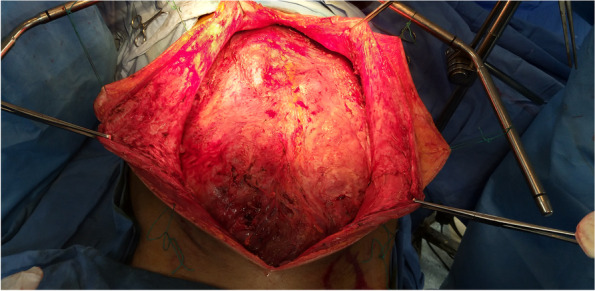
Extraperitoneal approach for CRS and HIPEC

We prefer an extraperitoneal approach with an incision from the xiphisternum to symphysis pubis under general anesthesia in a modified lithotomy position (Figs. 2, 3, 4). We perform radical appendicectomy, including removing lymph nodes caecectomy for clear margin or limited hemicolectomy as per the intraoperative assessment. No literature suggests that hemicolectomy yields better survival. We routinely remove all quadrant peritonectomy with an extraperitoneal approach, total greater and lesser omentectomy, and all the deposits over mesentery. Sometimes we perform partial gastrectomy, sigmoidectomy, and anterior resection to achieve complete CRS. We perform Gleason capsulectomy from the liver surface or by Hook technique to remove the scalloping over the liver and spleen. We sometimes use mucolytic agents like 5% dextrose and sodium bicarbonate. Other surgeons use hydrogen peroxide, ascorbic acid, N-acetyl cysteine, etc., as mucolytic agents to dissolve organized mucous. However, our experience may not be up to the mark, and more study will be required to optimize the potential agents, especially their dose and timing. In selected cases, some surgeons also perform CRS and HIPEC through a minimally invasive approach depending on the expertise.

**Fig. 5:**
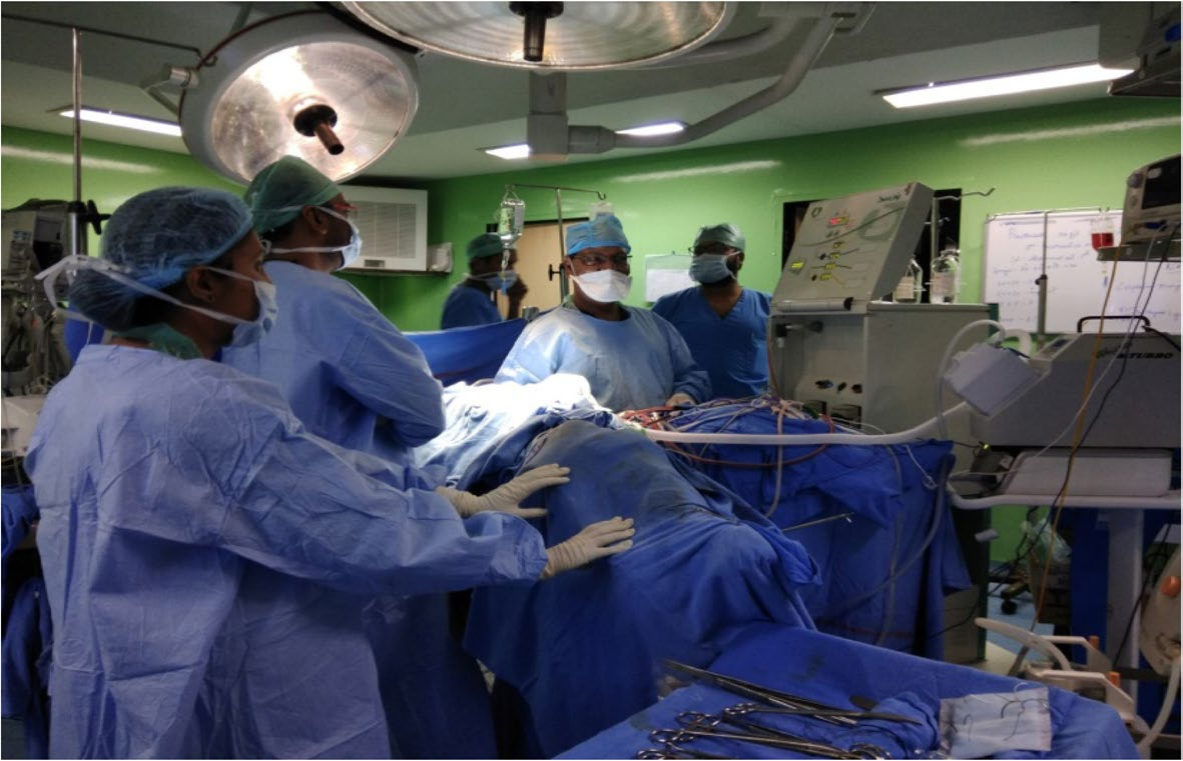
HIPEC in progress

In case of only limited acellular mucin with appendicular mass, we do appendicectomy with a clear margin with right lower quadrant peritonectomy, but in case of overspreading acellular mucin, we do total peritonectomy like the pattern of cellular mucin. In debulking surgery, we try to remove at least 90% of the tumor burden. We even perform CRS and HIPEC in case of gross and recurrent ascites for a better quality of life. We observed that in 90% of cases, pure ascites formation had been reduced after HIPEC. We prefer Doxorubicin 15mg/m^2^ in such a case along with Mitomycin C 15 mg/m2. In signet cell pathology, we perform CRS HIPEC only in well-differentiated histology and, to some extent, in moderately differentiated patients without any lymph nodes involvements only when complete CRS is achieved (CC-0). We use Mitomycin C 15mg/m^2^ or 40mg at 41–43 °C for 90 min (30mg at 1st hour and 10mg for the next half an hour). We usually perform HIPEC with a semi-open technique with a flow rate of 1000ml/min in our HIPEC machine (Fig. 5). We also perform repeat CRS or debulking surgery with or without HIPEC. As per the literature, despite CRS HIPEC, there is a significant recurrence of disease as high as 28% [32].

In post-operative follow-up, we evaluate tumor markers CEA, CA 19.9, and CA 125 every 3 months for the first 2 years, then every 6 months for the next 3 years, and annual screening with CECT scan imaging.

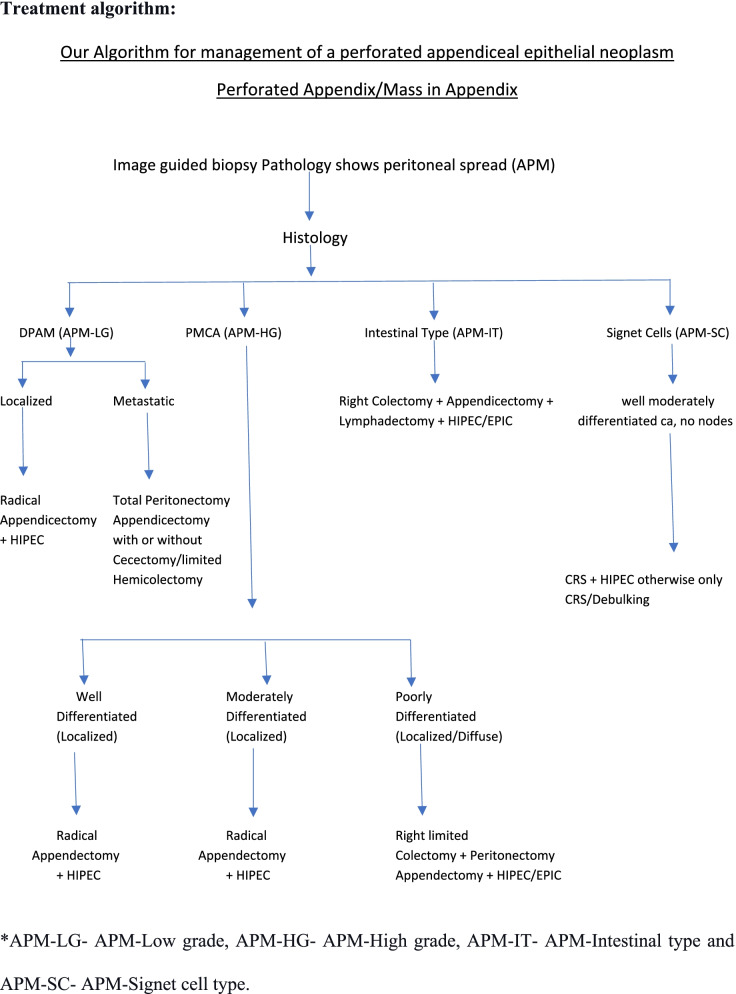



## Discussion

Controversy regarding the nomenclature of terminology has existed throughout the 137 years journey of PMP starting since 1884. Many advances have been made in understanding this disease's etiopathogenesis, natural history, histopathological, and management aspects. Still, the confusion persists, especially with the terms “pseudo” and “myxoma” in PMP. Today, several pieces of research have shown that these words bear no significance in compliance with the current understanding of the disease. To fix a unified nomenclature, we propose PMP as APM as the mucinous involvement is not only in the peritoneum but also involves other non-personalized abdominal organs. The term “mucinous carcinoma peritonei” is used by some authors, which also raises confusion as in a majority of cases, APM is indolent in nature and behaves like a benign disease. There is wide variation in nomenclature, and new classification systems still keep evolving. With the experience of our center and notable works from several other apex oncological centers across the globe, in today’s evidence-based era, it is high time to propose a new name of PMP to APM.

PMP is presently known as mucinous epithelial malignancy, originating from the appendix with a relatively broad spectrum of histologic differentiation. Aggressiveness is best manifested by its histological grading based on the limited pattern of morphologic criteria. Typically, most of our patients present between 45 and 66 years with a mean age of 55 years in our series. Most patients presenting to our center were referred cases from local hospitals with locally advanced and high disease burdens. Even the disease may be so indolent that it may go unnoticed for months or years without symptoms. The clinical presentation usually depends on the progression of the disease. The patients with localized disease may present as appendicitis or a pelvic mass. Patients may present with abdominal discomfort, distension due to mucinous ascites or bowel obstruction in more advanced disease. Another joint presentation is a new onset hernia due to increased intra-abdominal pressure. CECT is the standard imaging modality to assess for disease extent and resectability. The disease is best managed with aggressive CRS, which has a favorable impact on the overall survival of the patients. Better survival outcomes also necessitate the better selection of the patients and their proper rehabilitation before extensive surgery. The addition of intraperitoneal chemotherapy either intraoperatively or in the early post-operative period significantly improves the outcome of the condition. The patient should be preferably managed with a multimodality team at specialized centers with expertise in HIPEC. CRS combined with HIPEC is a complicated procedure associated with significant postoperative morbidity and mortality. Several studies have quoted variable morbidity rates between 33 to 56% and overall mortality rates ranging from 0 to 18% [33].

Genetic mutations and mucolytic agents are coming in a great way. Usually, the KRAS and GNAS mutations are commonly found in LAMN varieties. However, in high-grade cancers, P53 is more commonly mutated. Many mucolytic agents are being used to dissolve organized mucin like hydrogen peroxide, sodium bicarbonate, 5% dextrose, ascorbic acid, and N-acetyl cysteine. The correlation between expression of EGFR, MUC 7, MUC 20, and disease prognosis is yet to be evaluated.

Continuous data collection and analysis are needed worldwide to understand these rare tumors better. Because of the long latency period and indolent tumor behavior, extended follow-up of minimal 5 years is recommended, consisting of thorough physical examination, tumor markers, and imaging, to detect recurrences early.

### Why to remove the term “pseudo”

If we look back in the history, we could find out that PMP is a term of present-day controversies. If we consider the etymology of pseudomyxoma, we could avoid the controversies and fix a unified nomenclature. The word pseudomyxoma derives from the mucin-like substance in the peritoneal cavity termed pseudomucin. The term PMP was coined while a woman alleged to have a ruptured pseudomucinous cystadenoma of the ovary in 1884. However, today, we know that in most cases, the origin for PMP is an appendiceal neoplasm, often of low histological grade, and presently, ovarian tumors are hardly being considered a significant etiology of PMP. PMP classification is still under discussion, and experts’ panels still strive for consensus.

## Conclusion

With our long-term experience and advancement of scientific evidence, we like to propose a new name for PMP as APM. We believe that this paper will give a clear picture of this rare disease and standard management strategy without any confusion. The current sub-classification of PSOGI into LG-PMP, HG-PMP, and SC-PMP may, however, be re-termed as LG-APM, HG-APM, IT-APM, and SC-APM in future after this term is accepted. Anatomical location, node involvement, grade, patient’s performance status, completeness of CRS, and the experience of the center in HIPEC with skilled and dedicated surgeons are the primary determinant factors for the outcomes of the patients of APM.

## Data Availability

At the Department of Surgical Oncology, DR-BRA-IRCH, AIIMS, New Delhi, India.
